# Coronary physiology revisited

**DOI:** 10.1007/s12471-017-0982-3

**Published:** 2017-04-18

**Authors:** J. J. Piek

**Affiliations:** Department of Cardiology, Academic Medical Centre, Amsterdam, The Netherlands

Coronary angiography is still the gold standard in the diagnosis of coronary artery disease, although its limitations in the assessment of haemodynamic severity of epicardial stenosis are widely recognised [1, 2]. In the 1990s, these limitations of angiography led to the introduction of sensor-equipped guidewires measuring pressure and flow to optimise the diagnostic workup during cardiac catheterisation.

The most frequently used parameter is the fractional flow reserve (FFR), defined as the ratio of the pressure distal to a lesion relative to the aorta pressure during maximal hyperaemia. The concept of FFR assumes a minimal influence of the microvascular resistance during hyperaemic conditions, although a large variability of microvascular resistance exists even in patients with single vessel disease and a normal left ventricular function [3]. Moreover, this microvascular resistance is also influenced by numerous other factors like hypertension, diabetes, left ventricular hypertrophy and/or diffuse coronary artery disease.

The assessment of coronary flow reserve, defined as the ratio of the distal hyperaemic flow relative to baseline flow, may serve as an alternative. However, an accurate assessment of flow velocity is more cumbersome than FFR measurement. Moreover, the use of coronary flow reserve has also been criticised as it is determined by the resistance of the epicardial narrowing as well as the distal microvascular resistance and is therefore considered to be less lesion-specific.

The FAME trials have shown the usefulness of FFR and this parameter has emerged as a class I A indication for clinical decision-making in the absence of documentation of myocardial ischaemia. However, the use of FFR in cardiac catheterisation laboratories did not increase as rapidly as expected, despite its simplicity, which is partly explained by the time-consuming approach of using intravenous administration of hyperaemic agents. For this purpose, the instantaneous wave-free ratio (iFR), which assesses the diastolic pressure gradient during baseline conditions, was introduced. The major advantage of this approach is that it does not require hyperaemia and is therefore more suitable for daily clinical practice.

Two large clinical trials, DEFINE-FLAIR and SWEDEHEART, were designed as non-inferiority trials in a direct comparison between iFR and FFR for patient management using an iFR cut-off value of 0.89 and an FFR cut-off value of 0.8 [4, 5]. Both studies came to the same conclusion: iFR is non-inferior to FFR with respect to clinical outcome. The expectation is that the simple approach of iFR measurements will result in a warranted increase in the number of physiological assessments during cardiac catheterisation. This is important in view of the observed alterations in diagnostic workup for patients selected for coronary angiography. For the guidance of interventions, this clinical decision-making of coronary intervention should ideally be based upon an appropriate interpretation of the patient’s complaints, the results of non-invasive diagnostic stress tests, as well as the result of intracoronary haemodynamic measurements.

For many years now there has been a remarkable lack of documentation in records of myocardial ischaemia prior to cardiac catheterisation in daily clinical practice [6]. To guide coronary interventions in the absence of documentation, an operator depends upon the interpretation of complaints of the patients that are not always easy to judge, as well as the use of intracoronary haemodynamic parameters. The threshold for coronary angiography has been lowered even further after the introduction of CT scans as an alternative for the assessment of coronary artery disease and the abundance of patients with troponin-T elevation not associated with an acute coronary syndrome.

Against this background, it becomes more important to refine the current intracoronary haemodynamic techniques. Both iFR and FFR are pressure-derived parameters that are attractive because of their simplicity, but that do not take into account the complexity of coronary artery disease that we are confronted with in our daily clinical practice. For instance, the iFR does not require hyperaemia, but this parameter is characterised by a wide grey zone (0.86–0.93). For lesions within this grey zone it is advocated to use FFR as a next step in the diagnostic workup [7]. It is debatable whether this is the best approach, as an abnormal FFR (<0.80) may coincide with a normal coronary flow reserve (>2.0) because of non-flow limiting lesions (20% of the total amount of cases). These non-flow limiting lesions are not causing myocardial ischaemia and the prognosis is not distinctive from patients with normal haemodynamic characteristics, i. e. a normal FFR and a normal coronary flow reserve [8, 9]. Moreover, the use of combined pressure flow measurements in borderline cases does not only provide a coronary flow reserve, but it also generates a hyperaemic stenosis resistance index that is even more specific than FFR and coronary flow reserve [10].

The recognised shortcomings of pressure-derived parameters have led to a revival of flow sensor technology that is urgently needed to improve current intracoronary diagnostic techniques. In this respect, it is important to note that the patient, and not the diagnostic technique, should be considered as the gold standard. This means that the patient’s symptoms should improve if the diagnostic technique indicates that a coronary intervention is justified and the patient’s outcome should be safe if the test is normal. The publication of the DEFINE-FLAIR and the SWEDEHEART study is an essential step forward for the use of coronary physiology during cardiac catheterisation, but remember Churchill’s words: “Now, this is not the end. It is not even the beginning of the end. But it is, perhaps, the end of the beginning”.
